# Performance evaluation of a SARS-CoV-2 and influenza A/B combo rapid antigen test

**DOI:** 10.3389/fmolb.2024.1308202

**Published:** 2024-05-23

**Authors:** Kevin P. Rosenblatt, Hugo Romeu, Camille Romeu, Elder Granger

**Affiliations:** ^1^ Consultative Genomics, PLLC, Bellaire, TX, United States; ^2^ Healix Pathology, LLP, Bellaire, TX, United States; ^3^ RCE Group, Miami, FL, United States; ^4^ THE 5Ps, LLC, Centennial, CO, United States

**Keywords:** COVID-19, rapid antigen test, point-of-care testing, coronavirus, influenza

## Abstract

**Introduction:** The global COVID-19 pandemic and seasonal influenza outbreaks have drawn attention to the critical need for accurate and efficient diagnostic tools.

**Methods:** The performance of the InstaView COVID-19/Flu Ag Combo Test, which was designed to simultaneously detect the SARS-CoV-2, influenza A, and influenza B viruses, was analytically and clinically evaluated.

**Results:** The InstaView COVID-19/Flu Ag Combo Test exhibited robust detection capabilities, accurately identifying SARS-CoV-2, influenza A, and influenza B viruses over a wide concentration range (1.41 × 10^3^ to 7.05 × 10^4^ TCID50/mL). Extensive testing against potential cross-reactants and interferences yielded no false-positive results, indicating the high specificity of the test. Clinical evaluation further confirmed the kit’s reliability, with sensitivity ranging from 95.1% to 98.2% for SARS-CoV-2, 88.9%–95.2% for influenza A, and 91.7%–100% for influenza B depending on the sample type. The specificity was consistently 100% for all of the targeted viruses.

**Discussion:** The InstaView COVID-19/Flu Ag Combo Test thus demonstrated high performance, ease of use, rapid results, and the ability to precisely detect SARS-CoV-2 and influenza A/B infections, making it an effective tool in streamlining diagnostic workflows, optimizing resource allocation, and improving patient outcomes.

## Introduction

The COVID-19 pandemic has highlighted the crucial need for rapid and accurate diagnostic tools to identify and differentiate between common respiratory viral infections that overlap in their symptomatology. In addition to the SARS-CoV-2 virus responsible for COVID-19, influenza viruses, particularly influenza A and B, pose significant health risks, resulting in substantial morbidity and mortality worldwide. The clinical presentation of SARS-CoV-2 shares similarities with influenza illness, making it challenging to differentiate between the two infections based on symptom combinations. Distinguishing COVID-19 from influenza is important because, while they may have similar symptoms, they are treated with different medications, and patient management often, too, differs. A rapid test for differentiating influenza from COVID-19 at the point of contact between a healthcare provider and a patient is desired to facilitate a rapid response to illness be it patient isolation, hospitalization, or through medical management. The concurrent circulation of these two respiratory viruses further complicates clinical management, surveillance efforts, and public health interventions (Hills et al., 2020). Furthermore, distinguishing influenza A from B is important because the natural history of each disease is often different for Type B: influenza B tends to affect children more often and may also have a more serious outcome (Tran et al., 2016; Bhat, 2020).

Rapid antigen tests have emerged as valuable diagnostic tools because of their simplicity, speed, cost-effectiveness, and suitability for point-of-care settings (Peeling et al., 2021). These tests leverage specific antibodies to identify viral proteins, providing timely results that can aid in prompt decision-making for patient management, isolation strategies, and infection control measures. The development of a multiplexed antigen test capable of simultaneously detecting SARS-CoV-2 and influenza A/B viruses offers substantial advantages in terms of resource utilization, patient convenience, and diagnostic accuracy. However, at the time of our study, only a few multiplex rapid antigen tests that include simultaneous testing for influenza and SARS-CoV-2 had been approved for use in the United States (Table 4. Multiplex Assays Authorized for Simultaneous Detection of Influenza Viruses and SARS-CoV-2 by FDA, 2020).

Here, we present a comprehensive evaluation of a novel SARS-CoV-2 and influenza A/B combo rapid antigen test, the SG Medical, Inc. InstaView COVID-19/Flu Ag Combo test kit, which was designed to detect and differentiate between these viral pathogens rapidly and accurately. The SG Medical, Inc. InstaView COVID-19/Flu Ag Combo Testing Kit is intended to be used as a point-of-care (POC) testing product.

## Methods

### Ethics statement

The clinical evaluation described in this study was approved by the Advarra Institutional Review Board (Approval number: Tokimus-SG 3008 00023 MIA).

### Description of InstaView COVID-19/Flu Ag combo testing kit

The InstaView COVID-19/Flu Ag Combo test kit is a point-of-care, lateral flow immunoassay for determining the presence of SARS-CoV-2, influenza A, and influenza B antigens in human nasal and nasopharyngeal samples. The test device contains a nitrocellulose membrane strip that is pre-coated with anti-SARS-CoV-2, influenza A, and influenza B antibodies on the test line zone and goat anti-chicken IgY antibodies on the control line zone.

The InstaView COVID-19/Flu Ag Combo uses two anti-SARS-CoV-2 antibodies. Both anti-SARS-CoV-2 antibodies used in this product are monoclonal and available from Fapon Biotech Inc. (China). The clone number for the antibody on the conjugate pad antibody is 31F12 and the catalog number is 0638. The clone number for the antibody on the test line of the membrane is 31F11, and the catalog number is 0639. Both anti-SARS-CoV-2 antibodies used the same recombinant, full-length, nucleocapsid protein immunogen. The accession number is YP_009724397.2. For reference, the full-length sequence is provided below:

1 msdngpqnqr napritfggp sdstgsnqng ersgarskqr rpqglpnnta swftaltqhg

61 edlkfprgq gvpintnssp ddqigyyrra trrirggdgk mkdlsprwyf yylgtgpeag

121 ygankdgi iwvategaln tpkdhigtrn pannaaivlq lpqgttlpkg fyaegsrggs

181 qassrsssrs rnssrnstpg ssrgtsparm agnggdaala lllldrlnql eskmsgkgqq

241 qqgqtvtkks aaeaskkprq krtatkaynv tqafgrrgpe qtqgnfgdqe lirqgtdykh

301 wpqiaqfaps asaffgmsri gmevtpsgtw ltytgaikld dkdpnfkdqv illnkhiday

361 ktfpptepkk dkkkkadetq alpqrqkkqq tvtllpaadl ddfskqlqqs mssadstqa

The epitope recognized by the anti-SARS2-CoV-2 antibody (31F11) on the test line of the membrane consists of amino acids 44 through 175 (highlighted in green). The epitope recognized by the anti-SARS2-CoV-2 antibody (31F12) on the conjugate pad consists of amino acids 74 through 105 (highlighted in light blue).

**FIGURE 1:**
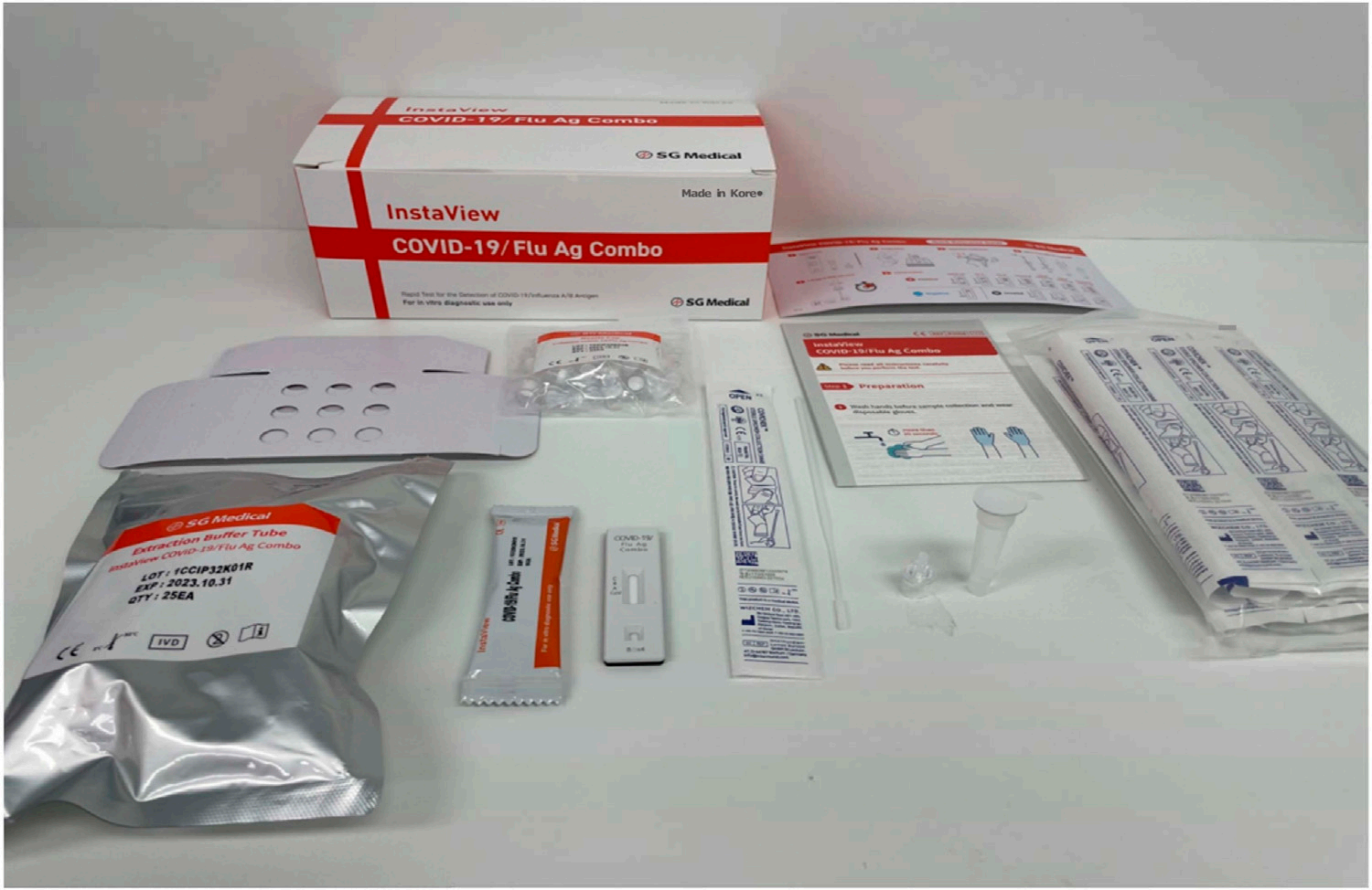
Picture of the InstaView COVID-19/Flu Ag combo test kit.

Neither the control line nor the test line is visible in the result window prior to application of the sample. A visible control line is required to indicate the test result is valid. When a patient’s sample is applied into the sample inlet, it migrates toward the conjugated pad that contains the anti-SARS-CoV-2, influenza A, or influenza B antibody-conjugated gold nanoparticles. When a sample contains SARS-CoV-2, influenza A, or influenza B antigens, antigen-antibody-gold complexes are formed. Here, the complexes flow along the nitrocellulose membrane, which is bound to the anti-SARS-CoV-2, influenza A or influenza B antibodies immobilized on the test line, by capillary action. While the sample migrates across the membrane along the strip, the chicken IgY-conjugated gold nanoparticles also move upward on the membrane chromatographically and bind to the anti-chicken IgY antibody immobilized on the control line. Red-colored lines become visible in the result window if SARS-CoV-2, Influenza A or Influenza B antigens are present in the sample (Figure 1). If there are no SARS-CoV-2, influenza A or influenza B antigens in the sample, no colored line appears on the test line, indicating a negative result. If a red colored line does not appear on the control line, the test is regarded as invalid.

### Limit of detection (LoD)

We determined the preliminary LoD of the InstaView COVID-19/Flu Ag Combo Test kit by testing a serial 10-fold dilutions series of inactivated culture fluid stocks prepared in a negative specimen. Each dilution was tested in 5 replicates. The lowest concentration at which all tested replicates were positive served as the preliminary LoD. The LoD was then confirmed by testing 20 individual replicates at 5 concentrations, including the preliminary LoD and concentrations above and below it. The confirmatory LoD was the lowest concentration resulting in positive detection of at least 95% of the replicates. This confirmatory study was performed with 2 replicates for each concentration for 5 days at two runs per day using three different lot numbers of product.

### Analytical reactivity (inclusivity)

The ability of the InstaView COVID-19/Flu Ag Combo Test kit to detect a variety of SARS-CoV-2, influenza A, and influenza B strains was evaluated. A total of five SARS-CoV-2 variants, alpha, beta, gamma, delta, and omicron, were included in the evaluation as well as H1N1, H1N1 pdm, H3N2, H5N1, H7N9, H9N2, and influenza B (Victoria and Yamagata). Contrived samples of all test strains were tested in 5 replicates in three-fold serial dilution from the stock samples. The lowest concentration of each strain that generated positive results in at least 1 of 5 replicates was determined to be the lowest detectable concentration.

### Cross-reactivity and microbial interference testing

We evaluated the potential cross-reactivity of the InstaView COVID-19/Flu Ag Combo test kit for the following organisms that may be encountered in nasopharyngeal swab specimens: *coronavirus HKU1*, *Staphylococcus epidermis*, Coronavirus SARS (SARS-CoV-1), *Mycoplasma pneumonia,* and *Chlamydia pneumonia*. Additionally, 41 potential interfering organisms, including adenovirus, *Bordetella pertussis*, *Candida albicans*, *Corynebacterium diphtheriae*, *Cytomegalovirus*, echovirus, enterovirus type 71, Epstein-Barr virus (EBV), *Escherichia coli*, *Haemophilus influenzae*, human adenovirus 1, human metapneumovirus 3 type B1, *Lactobacillus gasseri*, *Legionella pneumophila*, MERS-CoV, mumps virus, *Neisseria meningitides*, *Neisseria gonorrhoeae*, parainfluenza virus, pooled human nasal fluid, *Pseudomonas aeruginosa*, respiratory syncytial virus, rhinovirus, *Staphylococcus aureus*, *Streptococcus pneumoniae*, *Streptococcus pyogenes*, and *Streptococcus salivarius* were tested (Supplementary Appendix Table S1). To assess cross-reactivity, SARS-CoV, influenza A, and influenza B samples were spiked at concentrations close to the limit of detection (2X LoD) along with negative samples. The study included three replicates. To serve as a control, the same samples were tested without any cross-reactive samples.

### Clinical evaluation

An open-label prospective study was conducted to evaluate the clinical performance of the InstaView COVID-19/Flu A&B Combo (SG R3008) lateral flow rapid immunoassay in a study of rapid point-of-care testing for the COVID-19/FluA/FluB Combo using both nasopharyngeal and nasal swab specimens. Eligible subjects, including both symptomatic and asymptomatic individuals, who provided consent were enrolled in this “all-comer” type of study (see Supplementary Appendix for the inclusion/exclusion criteria). Enrollment continued until a minimum of 120 positive COVID-19 samples, 30 influenza A samples, and 20 influenza B samples and at least 200 negative samples were collected. The swab samples were then tested with the InstaView COVID-19/Flu Ag Combo (SG R3008), and the results were compared with the additional nasopharyngeal swabs collected from the subjects for PCR testing at the time of the rapid kit point-of-care testing using the reference method as the comparator (GenXPert XPress CoV-2/Flu/RSV plus).

## Results

We tested various SARS-CoV-2 variants, including alpha, beta, gamma and omicron. Details about the sequences of the detected viruses are provided in the Supplementary Appendix Table S1.

## Limit of detection (LoD)

The preliminary LoD of SARS-CoV-2 (isolate: USA-WA1/2020) was determined to be 1.41 × 10^5^ TCID_50_/mL, influenza A (H1N1 virus) as 1.41 × 10^3^ TCID_50_/mL, influenza A (H3N2 virus) as 1.51 × 10^3^ TCID_50_/mL, influenza B (strain: Victoria/504/00) as 1.41 × 10^4^ TCID_50_/mL, and influenza B (strain: Yamagata lineage) as 1.17 × 10^5^ TCID_50_/mL. By conducting further performance analysis, the LoD of SARS-CoV-2 (isolate: USA-WA1/2020) was confirmed as 7.05 × 10^4^ TCID_50_/mL, influenza A (H1N1 virus) as 1.41 × 10^3^ TCID_50_/mL, influenza B (H3N2 virus) as 1.51 × 10^3^ TCID_50_/mL, influenza B (strain: Victoria/504/00) as 1.41 × 10^4^ TCID_50_/mL influenza B (strain: Yamagata lineage) as 5.85 × 10^4^ TCID_50_/mL (Table 1).

**TABLE 1:** Limit of SARS-CoV-2, influenza A and influenza B antigen detection by the InstaView COVID-19/Flu Ag Combo test kit using SARS-CoV-2, influenza A, and influenza B culture fluid.

SARS-CoV-2 (isolate: USA-WA1/2020) culture fluid					
**Concentration (TCID_50_/mL)**	2.82 × 10^5^	1.41 × 10^5^	7.05 × 10^4^	3.53 × 10^4^	1.77 × 10^4^
Lot 1	20/20	20/20	20/20	2/20	0/20
Lot 2	20/20	20/20	20/20	3/20	0/20
Lot 3	20/20	20/20	20/20	2/20	0/20
Total (%)	60/60 (100%)	60/60 (100%)	60/60 (100%)	7/60 (11.7%)	0/60 (0%)
Influenza A H1N1 Culture Fluid (Isolate: New Caledonia/20/99)					
**Concentration (TCID**_**50**_ **/mL**)	2.82 × 10^3^	1.41 × 10^3^	7.05 × 10^2^	3.53 × 10^2^	1.77 × 10^2^
Lot 1	20/20	20/20	3/20	0/20	0/20
Lot 2	20/20	20/20	1/20	0/20	0/20
Lot 3	20/20	20/20	2/20	0/20	0/20
Total (%)	60/60 (100%)	60/60 (100%)	6/60 (10%)	0/60 (0%)	0/60 (0%)
Influenza A H3N2 Culture Fluid (Isolate: Texas/50/12)					
**Concentration (TCID**_**50**_ **/mL**)	3.02 × 10^3^	1.51 × 10^3^	7.55 × 10^2^	3.78 × 10^2^	1.89 × 10^2^
Lot 1	20/20	20/20	3/20	0/20	0/20
Lot 2	20/20	20/20	1/20	0/20	0/20
Lot 3	20/20	20/20	2/20	0/20	0/20
Total (%)	60/60 (100%)	60/60 (100%)	6/60 (10%)	0/60 (0%)	0/60 (0%)
Influenza B Culture Fluid (Isolate: Victoria Lineage/504/00)					
**Concentration (TCID**_**50**_ **/mL**)	2.82 × 10^4^	1.41 × 10^4^	7.05 × 10^3^	3.53 × 10^3^	1.77 × 10^3^
Lot 1	20/20	20/20	5/20	0/20	0/20
Lot 2	20/20	20/20	3/20	0/20	0/20
Lot 3	20/20	20/20	2/20	0/20	0/20
Total (%)	60/60 (100%)	60/60 (100%)	10/60 (16.7%)	0/60 (0%)	0/60 (0%)
Influenza B Culture Fluid (Isolate: Yamagata Lineage/16/88)					
**Concentration (TCID**_**50**_ **/mL**)	1.17 × 10^5^	5.85 × 10^4^	2.93 × 10^4^	1.46 × 10^4^	7.30 × 10^3^
Lot 1	20/20	20/20	4/20	0/20	0/20
Lot 2	20/20	20/20	3/20	0/20	0/20
Lot 3	20/20	20/20	4/20	0/20	0/20
Total (%)	60/60 (100%)	60/60 (100%)	11/60 (18.3%)	0/60 (0%)	0/60 (0%)

### Analytical reactivity (inclusivity)

An analytical reactivity study was performed to determine the ability of the InstaView COVID-19/Flu Ag Combo test kit to detect a variety of SARS-CoV-2, Influenza A and Influenza B strain that represent temporal and geographic diversity. The lowest concentration of each strain that generated positive results in at least 1 in 5 replicates is indicated in Table 2.

**TABLE 2:** Results of analytical reactivity: lowest detectable concentration for various SARS-CoV-2, influenza A, and influenza B strains.

Strain type	Strain name	Lowest detectable concentration
SARS-CoV-2 variant B1.1.7 USA/CA_CDC_5574/2020	SARS-CoV-2 alpha	4.32 × 10^3^ TCID_50_/mL
SARS-CoV-2 variant B.1.351 South-Africa/KRISP-K005325/202	SARS-CoV-2 beta	6.27 × 10^2^ TCID_50_/mL
SARS-CoV-2 variant Japan/TY7-503/2021	SARS-CoV-2 gamma	5.19 × 10^3^ TCID_50_/mL
SARS-CoV-2 variant B.1.617.2 USA/PHC658/2021	SARS-CoV-2 delta	5.19 × 10^3^ TCID_50_/mL
SARS-CoV-2 variant B1.1.529	SARS-CoV-2 omicron	6.87 × 10^2^ TCID_50_/mL
Influenza A (H1N1)	FM/1/47	1.40 × 10^6^ CEID_50_/mL
Influenza A (H1N1)	NWS/33	3.05 × 10^4^ CEID_50_/mL
Influenza A (H1N1)	Denver/1/57	2.19 × 10^6^ CEID_50_/mL
Influenza A (H1N1)	New Jersey/8/76	1.40 × 10^5^ CEID_50_/mL
Influenza A (H1N1)	Brisbane/59/2007	37.95 ngHA/mL
Influenza A (H1N1)	PR/8/34	7.00 × 10^2^ TCID_50_/mL
Influenza A (H1N1)	California/07/09	7.65 × 10 TCID_50_/mL
Influenza A (H1N1pdm)	Canada/6294/09	2.09 × 10^3^ TCID_50_/mL
Influenza A (H1N1pdm)	NY/02/09	5.19 × 10^3^ TCID_50_/mL
Influenza A (H1N1pfm)	Michigan/45/2015	44 ugHA/mL
Influenza A (H2N2)	Singapore/1/57	185.19 ngHA/mL
Influenza A (H3N2)	Port Chalmers/1/73	6.58 × 10^5^ CEID_50_/mL
Influenza A (H3N2)	Victoria/3/75	3.25 × 10^5^ CEID_50_/mL
Influenza A (H3N2)	New York/55/2004	679.01ngHA/mL
Influenza A (H3N2)	Hong Kong/2671/2019	2.70 ugHA/mL
Influenza A (H3N2)	Hong Kong/4801/2014	740.74 ngHA/mL
Influenza A (H3N2)	Brisbane/01/2018	5.56 ugHA/mL
Influenza A (H3N2)	Kansas/14/2017	827.16 ngHA/mL
Influenza A (H3N2)	Singapore/INFIMH-16-0019/2016	2.04 ugHA/mL
Influenza A (H3N2)	Hong Kong/8/68	2.07 × 10^3^ TCID_50_/mL
Influenza A (H3N2)	Wisconsin/67/05	1.72 × 10^3^ TCID_50_/mL
Influenza A (H3N2)	Perth/16/09	1.73 × 10^3^ TCID_50_/mL
Influenza A (H5N1)	Anhui/1/05	45.27 ngHA/mL
Influenza A (H7N9)	Anhui/1/2013	130.32 ngHA/mL
Influenza A (H9N2)	Chick/Hong Kong/G9/1997	115.23 ngHA/mL
Influenza B	Lee/40	1.98 × 10^4^ CEID_50_/mL
Influenza B	Malaysia/2506/04	5.22 × 10^3^ TCID_50_/mL
Influenza B	Allen/45	6.67 × 10^3^ CEID_50_/mL
Influenza B	GL/1739/54	5.93 × 10^6^ CEID_50_/mL
Influenza B	Taiwan/2/62	1.10 × 10^5^ CEID_50_/mL
Influenza B	Hong Kong/5/72	3.66 × 10^5^ CEID_50_/mL
Influenza B	Maryland/1/59	4.17 × 10^4^ CEID_50_/mL
Influenza B (Victoria Lineage)	Brisbane/60/2008	14 ugHA/mL
Influenza B (Victoria Lineage)	Washington/02/2019	25.33 ugHA/mL
Influenza B (Yamagata Lineage)	Phuket/3073/2013	1.05 × 10^6^ TCID_50_/mL

### Cross-reactivity and microbial interference testing

The results showed no cross-reactivity among the five potentially cross-reactive substances. The comparison of the results from the sample containing the cross-reactive/interfering substance and the control group showed identical results within the sample fit ranges. No false positive, false negative, or invalid results were observed. Additionally, none of the 41 organisms, including, adenovirus, *B. pertussis*, *C. albicans*, *C. diphtheriae*, *Cytomegalovirus*, echovirus, enterovirus type 71, Epstein-Barr virus (EBV), *E. coli*, *H. influenzae*, human adenovirus 1, human metapneumovirus 3 type B1, *L. gasseri*, *L. pneumophila*, MERS-CoV, mumps virus, *N. meningitides*, *N. gonorrhoeae*, parainfluenza virus, pooled human nasal fluid, *P. aeruginosa*, respiratory syncytial virus, rhinovirus, *S. aureus*, *S. pneumoniae*, *S. pyogenes*, and *Streptococcus salivarius *tested demonstrated any interference at the listed concentrations (Supplementary Appendix Table S1).

### Clinical evaluation

In the first cohort, 519 participants were available for analysis. Their average age was 64 years; 35% (n = 181) were male and 65% (n = 338) were female. A total of 62% (n = 322) of the individuals presenting for testing showed no discernible signs or symptoms of an upper respiratory infection. Of all the participants, 125 tested COVID-19 positive, 31 tested influenza A positive, and 21 tested influenza B positive using the comparator qRT-PCR method (Table 3). Among the age groups, the participants aged 40–64 years had the highest prevalence of COVID-19 (30%) while those >65 years had the highest prevalence of influenza A (8%), and those aged 2–14 years had the highest prevalence of influenza B (17%). The average Ct value of the participants testing COVID-19 positive was 26.2.

**TABLE 3:** Demographic distribution of the participants included in the clinical evaluation.

	Participants	COVID-19 positive (%)	Influenza A positive (%)	Influenza B positive (%)
Age (years)				
2-14	18	2 (11%)	0 (0%)	3 (17%)
15-39	64	17 (27%)	1 (2%)	2 (3%)
40-64	112	33 (30%)	4 (4%)	4 (4%)
≥65	325	73 (22%)	26 (8%)	12 (4%)
Sex				
Men	181	37 (20%)	7 (4%)	10 (6%)
Women	338	88 (26%)	24 (7%)	11 (3%)

For COVID-19, the overall sensitivity and specificity of the rapid antigen test were 96.8% (95%CI: 92.0%–99.1%) and 100% (95%CI: 98.9%–100%), respectively (Table 4). For nasal samples, the sensitivity and specificity were 98.3% (95%CI: 90.6%–100%) and 100% (95%CI: 97.6%–100%), respectively. For nasopharyngeal samples, the sensitivity and specificity were 95.1% (95%CI: 83.5%–99.4%) and 100% (95%CI: 97.5%–100%), respectively.

**TABLE 4:** Diagnostic accuracy of the InstaView COVID-19/Flu Ag Combo test in a real-life clinical setting. Sensitivities and specificities are in relation to RT-PCR and are given for the (a) overall study group as well as (b) samples from nasal swabs, (c) samples from nasopharyngeal swabs, and (d) samples that combined nasal and nasophayngeal swabs.

	COVID-19		Influenza A		Influenza B	
InstaView COVID-19/Flu Ag combo test results	RT-PCR positive	RT-PCR negative	RT-PCR positive	RT-PCR negative	RT-PCR positive	RT-PCR negative
Clinical evaluation for all samples overall						
Positive	121	0	29	0	20	0
Negative	4	342	2	342	1	342
Sensitivity	96.8% (95%CI: 92.0%–99.1%)		93.6% (95%CI: 78.6%–100%)		95.2% (95%CI: 76.2%–99.9%)	
Specificity	100% (95%CI: 98.9%–100%)		100% (95%CI: 98.9%–100%)		100% (95%CI: 98.9%–100%)	
Clinical evaluation for samples derived from nasal swabs						
Positive	56	0	8	0	6	0
Negative	1	150	1	150	0	150
Sensitivity	98.3% (95%CI: 90.6%–100%)		88.9% (95%CI: 51.8%–100%)		100% (95%CI: 54.1%–100%)	
Specificity	100% (95%CI: 97.6%–100%)		100% (95%CI: 97.6%–100%)		100% (95%CI: 97.6%–100%)	
Clinical evaluation for samples derived from nasopharyngeal swabs						
Positive	39	0	20	0	11	0
Negative	2	147	1	147	1	147
Sensitivity	95.1% (95%CI: 83.5%–99.4%)		95.2% (95%CI: 76.2%–100%)		91.2% (95%CI: 61.5%–100%)	
Specificity	100% (95%CI: 97.5%–100%)		100% (95%CI: 97.5%–100%)		100% (95%CI: 97.5%–100%)	
Clinical evaluation for samples that combined nasal and nasopharyngeal swabs						
Positive	26	0	1	0	3	0
Negative	1	45	0	45	0	45
Sensitivity	96.3% (95%CI: 81.0%–99.9%)		100% (95%CI: 2.5%–100%)		100% (95%CI: 29.2%–100%)	
Specificity	100% (95%CI: 92.1%–100%)		100% (95%CI: 92. 1%–100%)		100% (95%CI: 92. 1%–100%)	

For influenza A, the overall sensitivity and specificity of the rapid antigen test were 93.6% (95%CI: 78.6%–100%) and 100% (95%CI: 98.9%–100%), respectively (Table 4). The sensitivity of the test using nasal samples was 88.9% (95%CI: 51.8%–100%) and 95.2% (95%CI: 76.2%–100%) using nasopharyngeal samples. The specificity of the test using either sample type was 100% (95%CI: 97.6%–100%).

For influenza B, the overall sensitivity and specificity of the rapid antigen test samples were 95.2% (95%CI: 76.2%–99.9%) and 100% (95%CI: 99.3%–100%), respectively (Table 4). Sensitivity of the test using nasal samples was 100% (95%CI: 54.1%–100%) and 91.7% (95%CI: 61.5%–100%) using nasopharyngeal samples. The specificity of the test using either sample type was 100% (95%CI: 97.6%–100%).

## Discussion

In this study, we conducted a comprehensive performance evaluation of the InstaView COVID-19/Flu Ag Combo Test, which is capable of simultaneously detecting SARS-CoV-2, influenza A, and influenza B viruses. The ongoing global COVID-19 pandemic and seasonal influenza outbreaks have placed significant strain on healthcare systems (Simonsen et al., 2000; Bojdani et al., 2020; Moghadas et al., 2020), underscoring the need for accurate and efficient diagnostic tools to promptly identify and differentiate these respiratory infections. Timely identification and proper isolation and treatment of individuals with influenza-like illnesses are crucial for effectively managing respiratory infections and safeguarding healthcare infrastructure.

The test kits demonstrated the ability to accurately detect SARS-CoV-2, Influenza A and Influenza B viruses at concentrations ranging from 1.41 × 10^3^ TCID_50_/mL to 7.05 × 10^4^ TCID_50_/mL. The kit was challenged with a panel of potential cross-reactants and interferences but did not show any cross-reactivity or interference effects. This data indicates that the InstaView COVID-19/Flu Ag Combo Test is highly specific to the targeted viruses, reducing the likelihood of false-positive results. Clinical evaluation of the InstaView COVID-19/Flu Ag Combo Test kit demonstrated the high sensitivity and specificity of the kit in detecting SARS-CoV-2 and influenza A/B viruses. Depending on the sample type, sensitivity for SARS-CoV-2 ranged between 95.1% and 98.2%, 88.9%–95.2% for influenza A, and between 91.7% and 100% for influenza B. The specificity of the test kit was 100% for all viruses.

In conclusion, this kit demonstrated high sensitivity and specificity to SARS-CoV-2 and influenza viruses under rigorous analytical and clinical settings. The results exceeded the accuracy standards recommended in the guidelines of the FDA (Center for Devices and Radiological Health, 2023), WHO (Technical Specifications for Selection of Essential in vitro Diagnostics for SARS-CoV-2, 2021), and European Union (European Commission Directorate General for Health and Food Safety, 2022) regulatory agencies for COVID-19 antigen test kits. The performance of the InstaView device for COVID-19, including the high sensitivity, approaches that of many PCR-based assays and is higher than other rapid antigen tests for the same viruses. It is not readily clear why the clinical sensitivity is so high; it may be due to the affinities of the monoclonal antibodies to the antigens of the viruses used to make the monoclonal antibodies in the assays. Additionally, the detection chemistries appear to be very sensitive from a technical standpoint. High technical sensitivity in the assays would translate into a high clinical sensitivity if the device is better at ruling out the presence of a particular virus. A selective, specific, and high affinity antibody would be necessary for that purpose. The interference, exclusivity, and other analyses of the device demonstrate a high analytical sensitivity and specificity and support this possibility.

The findings of this evaluation have significant implications for clinical practice and public health surveillance, for detecting pandemic SARS-CoV-2 and influenza A/B infections using a single test could streamline diagnostic workflows, optimize resource allocation, and improve patient outcomes. Furthermore, the ability of the combo rapid antigen test to specifically detect SARS-CoV-2 and influenza A/B without cross-reactivity to other viruses will be a considerable advantage during the resurgence of other respiratory pathogens such as RSV and adenovirus.

## Data Availability

The original contributions presented in the study are included in the article/Supplementary Material, further inquiries can be directed to the corresponding author.
