# Supplementary material for: Performance evaluation of a SARS-CoV-2 and influenza A/B combo rapid antigen test
Source: Front Mol Biosci. 2024 May 23;11:1308202. doi: 10.3389/fmolb.2024.1308202 (PMC11154013; doi:10.3389/fmolb.2024.1308202)
Supplement: Supplementary file 1 [file Table1.DOCX]

**Performance evaluation of a SARS-CoV-2 and Influenza A/B combo rapid antigen test**

Kevin P. Rosenblatt^1,2*^ MD PhD, Hugo Romeu^3^ MD, Camille Romeu^3^ MD, Elder Granger^4^ MD

**Affiliations:**

1 Consultative Genomics, PLLC, 4800 Fournace Pace, Suite BE13, Bellaire, TX 77401

2 Healix Pathology, LLP, 4800 Fournace Place, Suite BW3, Bellaire, TX 77401

3 RCE Group, 25 SE 2nd Ave Suite 108, Miami, Fl 33131

4 MD, FACP, FACHE, MG, USA (retired) President/CEO THE 5Ps, LLC, SDVOSB, Healthcare, Thought Leadership, and Education Consulting

**Corresponding author:**

Kevin P. Rosenblatt, M.D., Ph.D.: Kevin.Rosenblatt@ConGenx.com; KR@healixpathology.com

**Inclusion/Exclusion criteria of Subjects for Clinical Trial:**

Inclusion Criteria:

1. Males or females, ages 2 and older with or without symptoms of Influenza or COVID-19.
2. Participant agrees to complete all aspects of the study.

Exclusion Criteria:

Subjects were excluded if any of the following exclusion criteria were present at the screening:

1. Consumption of food, liquids (except water), or alcohol within 30 minutes prior to first sample collection.
2. Use of any tobacco product within 30 minutes prior to first sample collection.
3. Any condition or finding that would put the subject or study conduct at risk if the subject were to participate in the study.
4. Medical professionals.
5. Employee of SG Medical, Inc., Tokimus, RCE, CHI, HCA, Heisu CRO
6. Subjects previously enrolled in the study

**Appendix Table 1: S**equences details of detected SARS-CoV-2 variants detected by InstaView COVID-19/Flu Ag Combo Test.


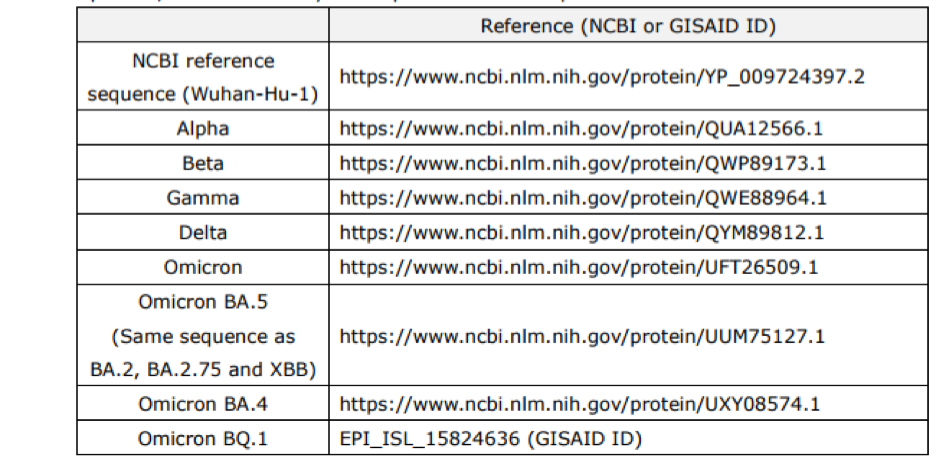


**Appendix Table 2:** Concentration at which the 41 tested substances demonstrated no interference

| Virus/Bacteria | Concentration |
| --- | --- |
| SARS-CoV-2 | 0.71X10^7^ TCID_50_/mL |
| Influenza A H1N1 Virus | 0.71X10^5^ TCID_50_/mL |
| Influenza B Virus | 0.71X10^5^ TCID_50_/mL |
| Adenovirus (Type3) | 1.70 X10^7^ TCID_50_/mL |
| Bordetella pertussis | 0.5 X10^6^ CFU/mL |
| Candida albicans | 0.5 X10^6^ CFU/m L |
| Coronavirus (Strain: 229E) | 2.08 X10^5^ TCID_50_/mL |
| Coronavirus (Strain: NL63) | 0.85 X10^5^ TCID_50_/mL |
| Coronavirus (Strain: OC43) | 2.50 X10^5^ TCID_50_/mL |
| Corynebacterium diphtheriae | 1.0 X10^10^ CFU/mL |
| Cytomegalovirus | 2,500 cp/mL |
| Echovirus | 0.5 X10^6^ TCID_50_/mL |
| Enterovirus Type 71 | 2. 8 X10^5^ TCID_50_/mL |
| Epstein-Barr Virus (EBV) | 1.40 X10^8^ cp/mL |
| Escherichia coli | 0.5 X10^10^ CFU/mL |
| Haemophilus influenzae | 0.5 X10^6^ CFU/mL |
| Human Adenovirus 1 | 0.7 X10^8^ PFU/mL |
| Human metapneumovirus 3 type B1  (Strain: Peru2-2002} | 0.7 X10^5^ TCID_50_/mL |
| Lactobacillus gasseri | 1.0 X10^9^ CFU/mL |
| Legionella pneumophila  (subse. Pneumophila) | 1.9 X10^6^ CFU/mL |
| Measles | 3.40 X10^6^ TCID_50_/mL |
| MERS-CoV  (Strain:Florida/USA-2_Saudi Arabia 2014) | 2.08 X10^5^ TCID_50_/mL |
| Moraxella catarrhalis | 0.5 X10^6^ CFU/m L |
| Mumps Virus | 0.57X10^7^ TCID_50_/mL |
| Neisseria meningitides | 0.5 X10^6^ CFU/mL |
| Neisseria gonorrhoeae | 0.5 X10^6^ CFU/mL |
| Parainfluenza virus type 1 | 0.63 X10^6^ TCID_50_/mL |
| Parainfluenza virus type 2 | 0.75 X10^6^ TCID_50_/mL |
| Parainfluenza virus type 3 | 1.69 X10^7^ TCID_50_/mL |
| Parainfluenza virus type 4A | 0.70 X10^5^ TCID_50_/mL |
| Parainfluenza virus type 4B | 1.09X 10^6^ PFU/mL |
| Pooled Human Nasal Fluid | 50% |
| Pseudomonas aeruginosa | 1.0 X10^10^ CFU/mL |
| Respiratory syncytial virus type A | 0.53 X10^6^ TCID_50_/mL |
| Respiratory syncytial virus type B | 1.2 X10^7^ PFU/mL |
| Rhinovirus | 1.07 X10^5^ TCID_50_/mL |
| Staphylococcus aureus | 0.5 X10^11^ CFU/mL |
| Streptococcus pneumoniae | 0.5 X10^7^ CFU/mL |
| Streptococcus pyogenes | 1.5 X10^8^ CFU/m L |
| Streptococcus salivarius | 5.0 X10^5^ CFU/mL |
| Adenovirus 7 | 2.0 X10^8^ PFU/mL |

**
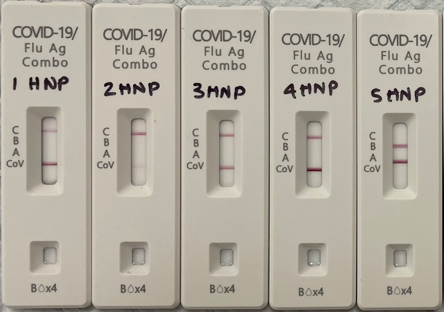
**

**Appendix Figure 1a*.*** Positive clinical COVID-19 Nasopharyngeal samples developed for 15 minutes. Four PCR-Confirmed Positive COVID-19 samples were Positive at the “CoV” Test Line. One patient sample ML8782, however, revealed Positive signals close to Test lines for “A” and “B” at 15 minutes, but no obvious band at the Control line indicated by “C.” Therefore, because that sample did not reveal a proper signal for the Control, it had to be discounted and is indicated as an “INVALID” result.

**
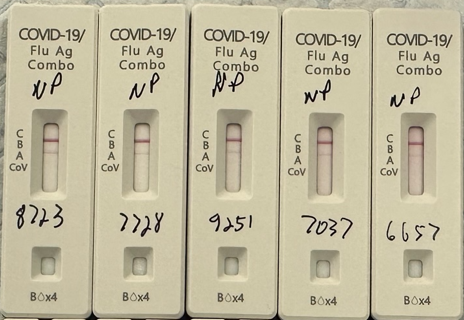
**

**Appendix Figure 1b*.*** Positive clinical Influenza B nasopharyngeal samples developed for 15 minutes. Five PCR-Confirmed Positive Flu B samples were Positive at the “B” Test Line. The lines were more prominent at 30 minutes of development (Data Not Shown), but the scoring of the Test Lines was prominent enough to record a sample as Positive by 15 minutes.

**
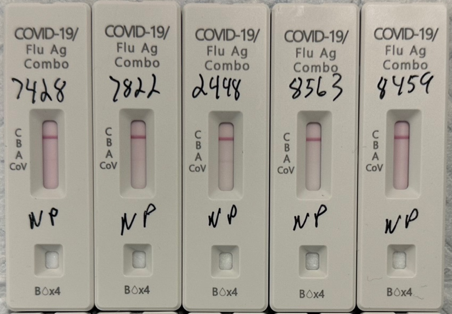
**

**Appendix Figure 2a*.*** Positive clinical Flu A Nasopharyngeal samples developed for 15 minutes. Five PCR-Confirmed Positive Flu A samples suggested Positive signals as faint bands at the “A” Test Line after 15 minutes wait time. As shown below in Figure 2, it took 35 minutes to develop to convincing bands at the “A” Test Lines. Note that 35 minutes of developing time is outside of the recommendations of the InstaView COVID-19/Flu Ag Combo kit IFU.

**
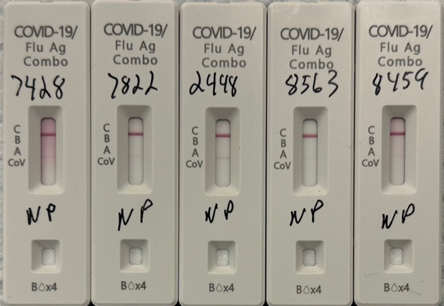
**

**Appendix Figure 2b*.*** Positive clinical Flu A Nasopharyngeal samples developed for 35 minutes. The Five (5) PCR-Confirmed Positive Flu A samples produced more intense Positive bands at the “A” Test Line at 35 minutes. The time-to-development was outside of the 30 minutes recommendations specified in the IFU.

**
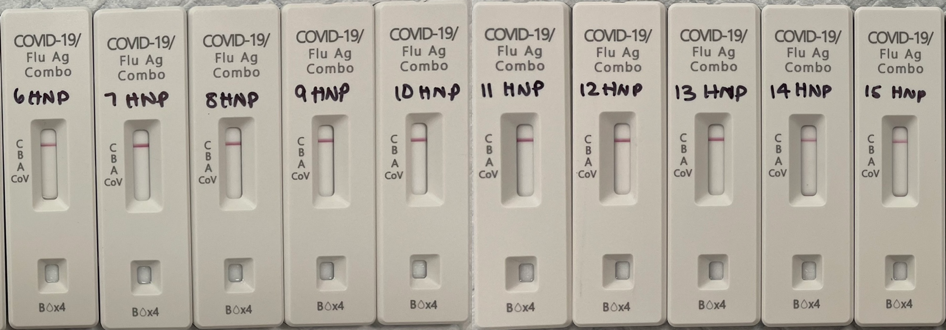
**

**Appendix Figure 3*.*** Negative clinical Nasopharyngeal samples developed for 30 minutes. All PCR Negative Nasopharyngeal samples were negative on InstaView after 30 minutes of development.
